# Adaptive design methods in clinical trials – a review

**DOI:** 10.1186/1750-1172-3-11

**Published:** 2008-05-02

**Authors:** Shein-Chung Chow, Mark Chang

**Affiliations:** 1 Duke University School of Medicine, Durham, North Carolina, USA; 2 Millennium Pharmaceuticals, Inc., Cambridge, Massachusetts, USA

## Abstract

In recent years, the use of adaptive design methods in clinical research and development based on accrued data has become very popular due to its flexibility and efficiency. Based on adaptations applied, adaptive designs can be classified into three categories: prospective, concurrent (ad hoc), and retrospective adaptive designs. An adaptive design allows modifications made to trial and/or statistical procedures of ongoing clinical trials. However, it is a concern that the actual patient population after the adaptations could deviate from the originally target patient population and consequently the overall type I error (to erroneously claim efficacy for an infective drug) rate may not be controlled. In addition, major adaptations of trial and/or statistical procedures of on-going trials may result in a totally different trial that is unable to address the scientific/medical questions the trial intends to answer. In this article, several commonly considered adaptive designs in clinical trials are reviewed. Impacts of ad hoc adaptations (protocol amendments), challenges in *by design *(prospective) adaptations, and obstacles of retrospective adaptations are described. Strategies for the use of adaptive design in clinical development of rare diseases are discussed. Some examples concerning the development of Velcade intended for multiple myeloma and non-Hodgkin's lymphoma are given. Practical issues that are commonly encountered when implementing adaptive design methods in clinical trials are also discussed.

## 1 Introduction

In the past several decades, it is recognized that increasing spending of biomedical research does not reflect an increase of the success rate of pharmaceutical (clinical) development. Woodcock (2005) indicated that the low success rate of pharmaceutical development could be due to (i) a diminished margin for improvement that escalates the level of difficulty in proving drug benefits, (ii) genomics and other new science have not yet reached their full potential, (iii) mergers and other business arrangements have decreased candidates, (iv) easy targets are the focus as chronic diseases are harder to study, (v) failure rates have not improved, (vi) rapidly escalating costs and complexity decreases willingness/ability to bring many candidates forward into the clinic [1]. As a result, the United States Food and Drug Administration (FDA) kicked off a *Critical Path Initiative *to assist the sponsors in identifying the scientific challenges underlying the medical product pipeline problems. In 2006, the FDA released a *Critical Path Opportunities List *that calls for advancing innovative trial designs, especially for the use of prior experience or accumulated information in trial design. The *Critical Path Opportunities List *interprets it as the encouragement for the use of innovative adaptive design methods in clinical trials and the potential use of Bayesian approach in clinical research and development. The purpose of adaptive design methods in clinical trials is to give the investigator the flexibility for identifying best (optimal) clinical benefit of the test treatment under study without undermining the validity and integrity of the intended study.

The concept of adaptive design can be traced back to 1970s when the adaptive randomization and a class of designs for sequential clinical trials were introduced [2]. As a result, most adaptive design methods in clinical research are referred to as adaptive randomization [3-6], group sequential designs with the flexibility for stopping a trial early due to safety, futility and/or efficacy [7-13], and sample size re-estimation at interim for achieving the desired statistical power [14-16]. The use of adaptive design methods for modifying the trial and/or statistical procedures of on-going clinical trials based on accrued data has been practiced for years in clinical research. Adaptive design methods in clinical research are very attractive to clinical scientists due to the following reasons. First, it reflects medical practice in real world. Second, it is ethical with respect to both efficacy and safety (toxicity) of the test treatment under investigation. Third, it is not only flexible, but also efficient in the early and late phase of clinical development. However, it is a concern whether the p-value or confidence interval regarding the treatment effect obtained after the modification is reliable or correct. In addition, it is also a concern that the use of adaptive design methods in a clinical trial may lead to a totally different trial that is unable to address scientific/medical questions that the trial is intended to answer [17,18].

In recent years, the potential use of adaptive design methods in clinical trials have attracted much attention. The Pharmaceutical Research and Manufacturers of America (PhRMA) and Biotechnology Industry Organization (BIO) have established adaptive design working groups and proposed strategies, methodologies, and implementations for regulatory consideration [19]. However, there are no universal agreement in terms of definition, methodologies, and applications. Many journals have published special sections and/or issues on adaptive design. These journals included, but are not limited to, Biometrics (Vol. 62, No. 3), Statistics in Medicine (Vol. 25, No. 19), Journal of Biopharmaceutical Statistics (Vol. 15, No. 4 and Vol. 17, No. 6), Biometrical Journal (Vol. 48, No. 4), and Pharmaceutical Statistics (Vol. 5, No. 2). For a comprehensive summarization of the issues and recommendations for the use of adaptive design methods, readers many consult with [18] and [20].

The purpose of this article is multi-fold. First, it is to provide a comprehensive review of adaptive design methods in clinical research and development. Second, it is not only to outline challenges in prospective adaptations, impact on concurrent adaptations, and obstacles of retrospective adaptations being made of on-going trials, but also to discuss issues that are commonly encountered when implementing adaptive design methods in clinical trials. Third, it is to discuss strategies for clinical development of rare diseases using adaptive design methods. In the next section, commonly employed adaptations and the resultant adaptive designs are briefly described. Also included in this section are regulatory and statistical perspectives regarding the use of adaptive design methods in clinical trials. The impact of protocol amendments, challenges of by design adaptations, and obstacles of retrospective adaptations when applying adaptive design methods in clinical trials are discussed in Section 3. In Section 4, strategies for the use of adaptive design in clinical development of rare diseases are discussed. Also included in this section are some trial examples concerning the development of Velcade (intended for multiple myeloma and non-Hodgkin's lymphoma). Practical issues that are commonly encountered when implementing adaptive design methods in clinical trials are discussed in Section 5. Some concluding remarks are given in the last section.

## 2 What is adaptive design?

In clinical trials, it is not uncommon to modify trial and/or statistical procedures during the conduct of clinical trials based on the review of interim data. The purpose is not only to efficiently identify clinical benefits of the test treatment under investigation, but also to increase the probability of success of clinical development. Trial procedures are referred to as the eligibility criteria, study dose, treatment duration, study endpoints, laboratory testing procedures, diagnostic procedures, criteria for evaluability, and assessment of clinical responses. Statistical procedures include randomization, study design, study objectives/hypotheses, sample size, data monitoring and interim analysis, statistical analysis plan, and/or methods for data analysis. In this article, we will refer to the adaptations (or modifications) made to the trial and/or statistical procedures as the adaptive design methods. Thus, an adaptive design is defined as a design that allows adaptations to trial and/or statistical procedures of the trial after its initiation without undermining the validity and integrity of the trial [21]. In their recent publication, with the emphasis of the feature of by design adaptations only (rather than ad hoc adaptations), the PhRMA Working Group on Adaptive Design refers to an adaptive design as a clinical trial design that uses accumulating data to decide on how to modify aspects of the study as it continues, without undermining the validity and integrity of the trial [19]. In many cases, an adaptive design is also known as a flexible design.

### 2.1 Adaptations

An adaptation is referred to a modification or a change made to trial and/or statistical procedure during the conduct of a clinical trial. By definition, adaptations that are commonly employed in clinical trials can be classified into the categories of prospective adaptation, concurrent (or ad hoc) adaptation, and retrospective adaptation. Prospective adaptations include, but are not limited to, adaptive randomization, stopping a trial early due to safety, futility or efficacy at interim analysis, dropping the losers (or inferior treatment groups), sample size re-estimation, and etc. Thus, prospective adaptations are usually referred to as by design adaptations as described in the PhRMA white paper [19]. Concurrent adaptations are usually referred to as any ad hoc modifications or changes made as the trial continues. Concurrent adaptations include, but are not limited to, modifications in inclusion/exclusion criteria, evaluability criteria, dose/regimen and treatment duration, changes in hypotheses and/or study endpoints, and etc. Note that concurrent adaptations are generally not envisioned as candidates for change initially but their needs become apparent as the trial continues. Retrospective adaptations are usually referred to as modifications and/or changes made to statistical analysis plan prior to database lock or unblinding of treatment codes. In practice, prospective, ad hoc, and retrospective adaptations are implemented by study protocol, protocol amendments, and regulatory reviewer's consensus, respectively.

### 2.2 Type of adaptive designs

Based on (primarily prospective) adaptations employed, commonly considered adaptive design methods in clinical trials include, but are not limited to: (i) an adaptive randomization design, (ii) a group sequential design, (iii) a sample size re-estimation design, (iv) a drop-the-loser design, (v) an adaptive dose finding (e.g., dose escalation) design, (vi) a biomarker-adaptive design, (vii) an adaptive treatment-switching design, (viii) a hypothesis-adaptive design, (ix) an adaptive seamless phase II/III trial design, and (x) a multiple adaptive design. These adaptive design methods are briefly described below.

#### Adaptive randomization design

**Figure 1:**
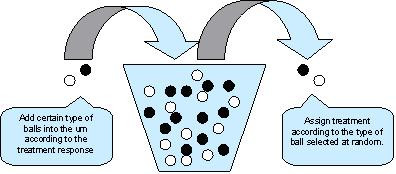
Response-adaptive randomization.

An adaptive randomization design is a design that allows modification of randomization schedules based on varied and/or unequal probabilities of treatment assignment in order to increase the probability of success (Figure 1) [See Additional file 1]. Commonly applied adaptive randomization procedures include treatment-adaptive randomization [3,4], covariate-adaptive randomization, and response-adaptive randomization [5,6]. Although an adaptive randomization design could increase the probability of success (so-called play-the-winner), it may not be feasible for a large trial or a trial with a relatively long treatment duration because the randomization of a given subject depends on the response of the previous subject. A large trial or a trial with a relatively long treatment duration utilizing adaptive randomization design will take a much longer time to complete. Besides, randomization schedule may not be available prior to the conduct of the study. Moreover, statistical inference on treatment effect is often difficult to obtain if it is not impossible due to complicated probability structure as the result of adaptive randomization.

#### Group sequential design

A group sequential design is a design that allows for prematurely stopping a trial due to safety, futility/efficacy or both with options of additional adaptations based on results of interim analysis. Various stopping boundaries based on different boundary functions for controlling an overall type I error rate are available in the literature (see, e.g., Lan and DeMets, 1987; Wang and Tsiatis, 1987; Rosenberger et al., 2001; Jennison and Turnbull, 2000, 2005; Chow and Chang, 2006). In recent years, the concept of two-stage adaptive design has led to the development of the adaptive group sequential design (e.g., Cui, Hung, and Wang, 1999; Posch and Bauer, 1999; Lehmacher and Wassmer, 1999; Liu, Proschan, and Pledger, 2002). It should be noted that the standard methods for group sequential design may not be appropriate (i.e., it may not be able to control the overall type I error rate at the desired level of 5%) if there is a shift in target patient population due to additional adaptations or protocol amendments.

#### Sample size re-estimation design

**Figure 2:**
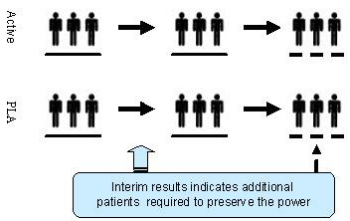
Sample size re-estimation.

A sample size re-estimation design is referred to as an adaptive design that allows for sample size adjustment or re-estimation based on the observed data at interim (Figure 2). Sample size adjustment or re-estimation could be done in either a blinding or unblinding fashion based on the criteria of treatment effect-size, conditional power, and/or reproducibility probability (see, e.g., [9,14-16], and [22-26]). Sample size re-estimation suffers from the same disadvantage as the original power analysis for sample size calculation prior to the conduct of the study because it is performed by treating estimates of the study parameters, which are obtained based on data observed at interim, as true values. It is not a good clinical/statistical practice to start with a small number and then perform sample size re-estimation (adjustment) at interim by ignoring the clinically meaningful difference that one wished to detect for the intended clinical trial. It should be noted that the observed difference at interim based on a small number of subjects may not be of statistically significance (i.e., it may be observed by chance alone). Thus, standard methods for sample size re-estimation based on the observed difference with a limited number of subjects may be biased and misleading.

#### Drop-the-losers design

A drop-the-losers design is a design that allows dropping the inferior treatment groups. A drop-the-losers design also allows adding additional arms. A drop-the-losers design is useful in phase II clinical development especially when there are uncertainties regarding the dose levels [27-30]. The selection criteria and decision rules play important roles for drop-the-loser designs. Dose groups that are dropped may contain valuable information regarding dose response of the treatment under study. Typically, drop-the-loser design is a two-stage design. At the end of the first stage, the inferior arms will be dropped based on some pre-specified criteria. The winners will then proceed to the next stage. In practice, the study is often powered for achieving a desired power at the end of the second stage (or at the end of the study). In other words, there may not be any statistical power for the analysis at the end of the first stage for dropping the losers (or picking up the winners). In this case, it is a common practice to drop the losers or pick up the winners based on so-called precision analysis, i.e., an approach for determining the confidence level for achieving a statistical significance. Note that some clinical scientists prefer the term *pick-the-winner designs*.

#### Adaptive dose finding design

An adaptive dose finding (e.g., escalation) design is often used in early phase clinical development to identify the minimum effective dose (MED) and/or the maximum tolerable dose (MTD), which is used to determine the dose level for the next phase clinical trials [31-33]. For adaptive dose finding design, the method of continual re-assessment method (CRM) in conjunction with Bayesian approach is usually considered [34-36]. Mugno et al. (2004) introduced a nonparametric adaptive urn design approach for estimating a dose-response curve [37]. More details regarding PhRMA's current position and proposed statistical methods, the reader should consult with a special issue recently published at the *Journal of Biopharmaceutical Statistics*, Vol. 17, No. 6.

#### Biomarker-adaptive design

A biomarker-adaptive design is a design that allows for adaptations based on the response of biomarkers such as genomic markers. An-adaptive biomarker design involves biomarker qualification and standard, optimal screening design, and model selection and validation. It should be noted that there is a gap between identifying biomarkers that associated with clinical outcomes and establising a predictive model between relevant biomarkers and clinical outcomes in clinical development. For example, correlation between biomarker and true clinical endpoint makes a prognostic marker. However, correlation between biomarker and true clinical endpoint does not make a predictive biomarker.

*A prognostic biomarker *informs the clinical outcomes, independent of treatment. They provide information about the natural course of the disease in individuals who have or have not received the treatment under study. Prognostic markers can be used to separate good- and poor-prognosis patients at the time of diagnosis. *A predictive biomarker *informs the treatment effect on the clinical endpoint ([20]).

A biomarker-adaptive design can be used to (i) select right patient population (e.g., enrichment process for selection of a better target patient population), (ii) identify nature course of disease, (iii) early detection of disease, and (iv) help in developing personalized medicine (see, e.g., [20] and [38,39]).

#### Adaptive treatment-switching design

An adaptive treatment-switching design is a design that allows the investigator to switch a patient's treatment from an initial assignment to an alternative treatment if there is evidence of lack of efficacy or safety of the initial treatment (see, e.g., [36] and [40]). In oncology clinical trials, estimation of survival is a challenge when treatment-switching has occurred in some patients. A high percentage of subjects who switched due to disease progression could lead to change in hypotheses to be tested. In this case, sample size adjustment for achieving a desired power is necessary.

#### Adaptive-hypotheses design

An adaptive-hypotheses design refers to a design that allows modifications or changes in hypotheses based on interim analysis results [41]. Adaptive-hypotheses designs often considered before database lock and/or prior to data unblinding. Some examples include the switch from a superiority hypothesis to a non-inferiority hypothesis and the switch between the primary study endpoint and the secondary endpoints. For the switch from a superiority hypothesis to a non-inferiority hypothesis, the selection of non-inferiority margin is critical which has an impact on sample size adjustment for achieving the desired power. According to the ICH guideline, the selected non-inferiority margin should be both clinical and statistical justifiable.

#### Adaptive seamless phase II/III design

**Figure 3:**
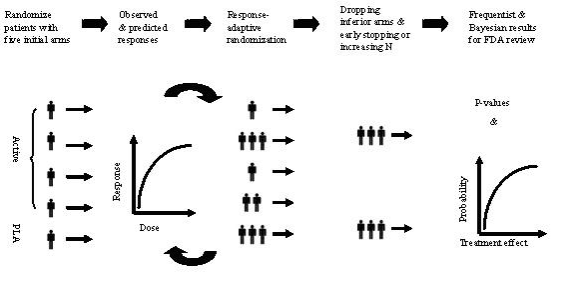
Phase II/III seamless trial design.

An adaptive seamless phase II/III trial design is referred to a program that addresses within single trial objectives that are normally achieved through separate trials in phase IIb and phase III of clinical development. An adaptive seamless phase II/III design (Figure 3) is an adaptive seamless phase II/III trial design that would use data from patients enrolled before and after the adaptation in the final analysis [42,43]. An adaptive seamless phase II/III design is a two-stage design consisting of a so-called learning stage (phase IIb) and a confirmatory stage (phase III). A typical approach is to power the study for the phase III confirmatory phase and obtain valuable information with certain assurance using confidence interval approach at the phase II learning stage. Its validity and efficiency, however, has been challenged [44]. Moreover, it is not clear how to perform a combined analysis if the study objectives (or endpoints) are similar but different at different phases [45]. More research is needed.

#### Multiple adaptive design

Finally, a multiple-adaptive design is any combinations of the above adaptive designs. Commonly considered multiple-adaptation designs include (i) the combination of adaptive group sequential design, drop-the-losers design, and adaptive seamless trial design and (ii) adaptive dose-escalation design with adaptive randomization [18]. In practice, since statistical inference for a multiple-adaptation design is often difficult, it is suggested that a clinical trial simulation be conducted to evaluate the performance of the resultant multiple adaptive design at the planning stage.

### 2.3 Statistical and regulatory perspectives

From regulatory point of view, the use of adaptive design methods based on accrued data in clinical trials may introduce operational bias such as selection bias, or bias that results from the method of evaluation, early withdrawal, and modification of treatment. Consequently, it may not be able to preserve the overall type I error rate at the pre-specified level of significance. In addition, the naive p-values may not be correct and the corresponding confidence intervals for the treatment effect may not be reliable. Moreover, it may result in a totally different trial that is unable to address the medical questions that original study intended to answer. In practice, commonly seen adaptations include, but are not limited to, (i) sample size adjustment at interim, (ii) sample size allocation to treatments, (iii) delete, add, or change treatment arms, (iv) shift in target patient population such as changes in inclusion/exclusion criteria, (v) change in statistical test strategy, (vi) change in study endpoints, and (vii) change in study objectives such as the switch from a superiority trial to a non-inferiority trial. One of the drawbacks of adaptive designs is that interpretation of trial results is difficult, such as the clinically meaningful effect size for the treatments under study [46].

From statistical point of view, major (or significant) adaptations to trial and/or statistical procedures could (i) introduce bias/variation to data collection, (ii) result in a shift in location and scale of the target patient population, and (iii) lead to inconsistency between hypotheses to be tested and the corresponding statistical tests. These concerns will not only have an impact on the accuracy and reliability of statistical inference drawn on the treatment effect, but also present challenges to biostatisti-cians for development of appropriate statistical methodology for an unbiased and fair assessment of the treatment effect.

Note that although the flexibility of modifying study parameters is very attractive to clinical scientists, several regulatory questions/concerns arise. First, what level of modifications to the trial procedures and/or statistical procedures would be acceptable to the regulatory authorities? Second, what are the regulatory requirements and standards for review and approval process of clinical data obtained from adaptive clinical trials with different levels of modifications to trial procedures and/or statistical procedures of on-going clinical trials? Third, has the clinical trial become a totally different clinical trial after the modifications to the trial procedures and/or statistical procedures for addressing the study objectives of the originally planned clinical trial? These concerns should be addressed by the regulatory authorities before the adaptive design methods can be widely accepted in clinical research and development.

## 3 Impact, challenges, and obstacles

### 3.1 Impact of protocol amendments

In practice, for a given clinical trial, it is not uncommon to have 3–5 protocol amendments after the initiation of the clinical trial. One of the major impacts of many protocol amendments is that the target patient population may have been shifted during the process, which may have resulted in a totally different target patient population at the end of the trial. A typical example is the case when significant modifications are applied to inclusion/exclusion criteria of the study protocol. As a result, the resultant actual patient population following certain modifications to the trial procedures is a *moving *target patient population rather than a fixed target patient population. As indicated in [18], the impact of protocol amendments on statistical inference due to shift in target patient population (moving target patient population) can be studied through a model that link the moving population means with some covariates [47]. Chow and Shao (2005) derived statistical inference for the original target patient population for simple cases [46].

### 3.2 Challenges in *by design *adaptations

In clinical trials, commonly employed prospective (by design) adaptations include stopping the trial early due to safety, futility, and/or efficacy, sample size re-estimation (adaptive group sequential design), dropping the losers (adaptive dose finding design), and combining two separate trials into a single trial (adaptive seamless design). These designs are typical multiple-stage designs with different adaptations. In this section, major challenges in analysis and design are described. Recommendations and future development for resolution are provided whenever possible.

The major difference between a classic multiple-stage design and an adaptive multiple-stage design is that an adaptive design allows adaptations after the review of interim analysis results. These *by design *adaptations include sample size adjustment (re-assessment or re-estimation), stopping the trials due to safety, efficacy/futility, or dropping the losers (picking the winners). Note that commonly considered adaptive group sequential design, adaptive dose finding design, and adaptive seamless trial design are special cases of multiple-stage designs with different adaptations. In this section, we will discuss major challenges in design (e.g., sample size calculation) and analysis (controlling type I error rate under moving target patient population) of an adaptive multiple-stage design with *K *– 1 interim analyses.

A multiple-stage adaptive group sequential design is very attractive to sponsors in clinical development. However, major (or significant) adaptations such as modification of doses and/or change in study endpoints may introduce bias/variation to data collection as the trial continues. To account for these (expected and/or unexpected) biases/variation, statistical tests are necessary adjusted to maintain the overall type I error and the related sample size calculation formulas have to be modified for achieving the desired power. In addition, the impact on statistical inference is not negligible if the target patient population has been shifted due to major or significant adaptations and/or protocol amendments. This has presented challenges to biostatisticians in clinical research when applying a multiple-stage adaptive design. In practice, thus, it is worthy pursuing the following specific directions that (i) deriving valid statistical test procedures for adaptive group sequential designs assuming model, which relates the data from different interim analyses, (ii) deriving valid statistical test procedures for adaptive group sequential designs assuming the random-deviation model, (iii) deriving valid Bayesian methods for adaptive group sequential designs, and (iv) deriving sample size calculation formulas for various situations. Tsiatis and Mehta (2003) showed that there exists an optimal (i.e., uniformly more powerful) design for any class of sequential design with a specified error spending function. It should be noted that adaptive designs do not require in general a fixed error spending function [44]. One of major challenges for an adaptive group sequential design is that the overall type I error rate may be inflated when there is a shift in target patient population [48].

For adaptive dose finding design, Chang and Chow's method can be improved by the following specific directions that (i) studying the relative merits and disadvantage of their method under various adaptive methods, (ii) examining the performance of an alternative method by forming the utility first with different weights to the response levels and then modeling the utility, and (iii) deriving sample size calculation formulas for various situations. An adaptive seamless phase II/III design is a two-stage design that consists of two phases namely a learning (or exploratory) phase and a confirmatory phase. One of the major challenges for designs of this kind is that different study endpoints are often considered at different stages for achieving different study objectives. In this case, the standard statistical methodology for assessment of treatment effect and for sample size calculation cannot be applied.

For a two-stage adaptive design, the above described method by Chang (2007b) can be applied [49]. Chang's method, however like other stage-wise combination methods, is valid under the assumption of constancy of the target patient populations, study objectives, and study endpoints at different stages.

### 3.3 Obstacles of retrospective adaptations

In practice, retrospective adaptations such as adaptive-hypotheses may encounter prior to database lock (or unblinding) and implemented through the development of statistical analysis plan. To illustrate the impact of retrospective adaptations, we consider the situation where switching hypotheses between a superiority hypothesis and a non-inferiority hypothesis. For a promising test drug, the sponsor would prefer an aggressive approach for planning a superiority study. The study is usually powered to compare the promising test drug with an active control agent.

However, the collected data may not support superiority. Instead of declaring the failure of the superiority trial, the sponsor may switch from testing superiority to testing the following non-inferiority hypotheses. The margin is carefully chosen to ensure that the treatment of the test drug is larger than the placebo effect and, thus, declaring non-inferiority to the active control agent means that the test drug is superior to the placebo effect. The switch from a superiority hypothesis to a non-inferiority hypothesis will certainly increase the probability of success of the trial because the study objective has been modified to establishing non-inferiority rather than showing superiority. This type of switching hypotheses is recommended provided that the impact of the switch on statistical issues and inference (e.g., appropriate statistical methods) on the assessment of treatment effect is well justified.

## 4 Strategies for clinical development of rare diseases

### 4.1 Adaptive design strategies

Clinical development of a new drug product is a lengthy and costly process, which includes phases I to III clinical development (prior to regulatory review and approval) and phase IV clinical development (post-approval). For life-threatening diseases or diseases with unmet medical need (rare diseases), this lengthy clinical development process is not acceptable. Thus, the use of adaptive design methods in clinical trials is called for. The purpose is to shorten the development process (speed) without compromising the safety and efficacy of the drug product under investigation (validity) by maximizing the power for identify best clinical benefit of the drug product under investigation with limited number of subjects (efficiacy). As a result, many adaptive design methods are developed for archiving the ultimate goals of validity, efficiency, and speed in clinical development. In practice, it however should be noted that many other important factors beyond statistical components may have an impact on the development process. These factors include, but are not limited to, patient enrollment, treatment durations, and time required for regulatory review/approval.

Commonly considered strategies for the use of adaptive design methods in clinical development include, but are not limited to, adaptive dose finding, adaptive seamless phase I/II in early clinical development, and adaptive seamless phase II/III in late phase clinical development. These strategies help in not only shortening the development time in a more efficient way but also increasing the probability of success in clinical development. As an example, let us consider the development of a new drug product for a rare disease of multiple myeloma (MM) with unmet medical need. A traditional approach is to conduct a typical phase I study for identifying the maximum tolerated dose (MTD), which is often considered as the optimal dose for the late phase of clinical development. In practice, there are several options that we can run this study with several doses or dose schedules. For example, this trial can be run with parallel dose groups or sequentially with the test drug as a single or add-on agent. The traditional approach will not be able to provide the entire spectrum of the dose response of the test drug. On the other hand, if we consider an adaptive approach, it allows us to use more options at the beginning and drop some of the arms (options) if they are either too toxic or ineffective (activity too low or requires very high dose that makes the treatment very costly biologic product may be very costly). In addition, since the treatment arms are often correlated, the information such as dose limiting toxicity (DLT) from different arms can be synchronized using methods such as Bayesian model [36].

Similar approaches can be used for phase II trials, phase I/II, or phase II/III adaptive seamless design. In this approach, the interim endpoint may be a marker such as response rate or time to disease progression. Some of the inferior arms can be dropped at interim. If some of the arms are promising, we can apply different randomization scheme to assign more patients to the superior arms (play-the-winner). In this case, not only the total cost will not increase, but also we will increase the chance of success because the increase of sample size for the superior treatment arms are most promising. In addition, the schedules for the trial are similar because the total number of patients remains unchanged. This adaptive design strategy can be applied to phase III trials, but with much less arms. For phase IV studies, the adaptive design can also be applied. However, the situation is different because the drug has been approved during phase IV clinical development. The focus of phase IV clinical development will be the safety rather than efficacy.

In what follows, adaptive design strategies are illustrated through some trial examples regarding the development of the drug product intended for the rare disease of multiple myeloma. Without loss of generality, data are modified from actual trials for the illustration purpose.

### 4.2 Some trial examples

#### Example 1: Dose-finding (escalation) design

In a phase I oncology trial, suppose that the primary objective is to identify the maximum tolerated dose (MTD) for a new test drug. Based on the animal studies, it is estimated that the toxicity (dose limiting toxicity rate) is 1% for the starting dose 25 mg/m^2 ^(1/10 of the lethal dose). The dose limiting toxicity (DLT) rate at MTD is defined as 0.25 and the MTD is estimated to be 150 mg/m^2^. A typical approach is to consider so-called 3+3 traditional escalation rule (TER). On the other hand, an adaptive type approach is to apply the continual re-assessment method (CRM) in conjunction with a Bayesian approach. We can compare the 3+3 TER approach and the Bayesian CRM adaptive method through simulations. A logistic toxicity model is chosen for the simulations. The dose interval sequence is chosen to be the customized dose increment sequence (Increment factors = 2, 1.67, 1.33, 1.33, 1.33, 1.33, 1.33, 1.33, 1.33). The evaluations of the escalation designs are based on the criteria of safety, accuracy, and efficiency. Simulation results for all three methods for three different MTD scenarios are summarized in Table 1.

**Table 1:** Summary of simulation results for the designs

Method	Assumed True MTD	Mean Predicted MTD	Mean Number of Patient	Mean Number of DLTs
3+3 TER	100	86.7	14.9	2.8
CRM	100	99.2	13.4	2.8
3+3 TER	150	125	19.4	2.9
CRM	150	141	15.5	2.5
3+3 TER	200	169	22.4	2.8
CRM	200	186	16.8	2.2

In this example, as it can be seen that if the true MTD is 150 mg/m^2^; the TER approach underestimates MTD (125 mg/m^2^), while the Baysian CRM adaptive method also slightly underestimates the MTD (141 mg/m^2^). The average number of patients required is 19.4 and 15.5 for the TER approach and the Bayesian CRM adaptive method, respectively. From a safety perspective, the average number of DLTs is 2.9 and 2.5 per trial for the TER approach and the Bayesian CRM, respectively. As a result, the Bayesian CRM adaptive method is preferable.

#### Example 2: Three-stage adaptive design for NHL trial

**Figure 4:**
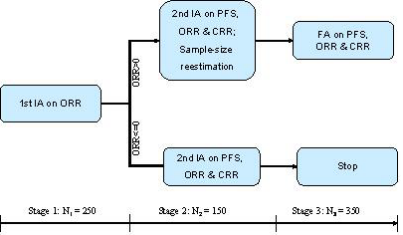
Non-Hodgkin's lymphoma trial.

A phase III two parallel group Non-Hodgkin's Lymphoma trial was designed with three analyses. The primary endpoint is progression-free survival (PFS), the secondary endpoints are (1) overall response rate (ORR) including complete and partial response and (2) complete response rate (CRR). The estimated median PFS is 7.8 months and 10 months for the control and test groups, respectively. Assume a uniform enrollment with an accrual period of 9 months and a total study duration of 23 months. The estimated ORR is 16% for the control group and 45% for the test group. The classic design with a fixed sample-size of 375 subjects per group will allow for detecting a 3-month difference in median PFS with 82% power at a one-sided significance level of α = 0:025: The first interim analysis (IA) will be conducted on the first 125 patients/group (or total *N*_1 _= 250) based on ORR. The objective of the first IA is to modify the randomization. Specifically, if the difference in ORR (test-control), Δ_*ORR *_> 0, the enrollment will continue. If Δ_*ORR *_≤ 0, then the enrollment will stop. If the enrollment is terminated prematurely, there will be one final analysis for efficacy based on PFS and possible efficacy claimed on the secondary endpoints. If the enrollment continues, there will be an second interim analysis based on PFS. The second interim analysis will could lead to either claim efficacy or futility, or continue to the next stage with possible sample-size reestimation. The final analysis of PFS. When the primary endpoint (PFS) is significant, the analyses for the secondary endpoints will be performed for the potential claim on the secondary endpoints. During the interim analyses, the patient enrollment will not stop. This example is modified slightly from an ongoing adaptive trial (Figure 4).

#### Example 3: Two-stage drop-loser adaptive design for optimal treatment in a phase IV trial

For phase IV study, the adaptive design can also work well. For example, after a year since an oncology drug in the market, physicians have used the drug with different combinations for treating patients with MM. However, there is a strong desire to know which combination is the best for the patient populations. Many physicians have their own experiences, no one has convincing data. Therefore, the sponsor is planned a trial to investigate the optimal combination for the drug. In this scenario, we can use much smaller sample size than phase III trials because the issue is not focused on the type-I control as the drug is approved. The issue is that given a minimum clinically meaningful difference (e.g., two weeks in survival), what is the probability of the trial will be able to identify the optimal combination. Again this can be done through simulations, the strategy is to start with about five combinations, drop inferior arms and calculate the probability of selecting the best arm under different combinations of sample size at the interim and final stages. In this adaptive design, we will drop 2 arms based on the observed response rate, i.e., the two arms with lowest observed response rates will be dropped at interim analysis and the rest three arms will be carried forward to the second stage. The characteristics of the design are presented in Table 2 for different sample sizes.

**Table 2:** Simulation results of phase IV drop-the-loser design

Interim sample size per group	Final sample size per group	Probability of identifying the optimal arm
25	50	80.3%
50	100	91.3%

Note: response rate for the five arms: 0.4, 0.45, 0.45, 0.5, and 0.6.

Given the response rate 0.4, 0.45, 0.45, 0.5, and 0.6 for the five arms and 91% power, if a traditional design is used, there will be 9 multiple comparisons (adjusted alpha = 0.0055 using Bonferroni method). The required sample size for the traditional design is 209 per group or a total of 1045 subjects comparing a total of 500 subjects for the adaptive trial (250 at the first stage and 150 at the second stage). In the case when the null hypothesis is true (the response rates are the same for all the arms), it doesn't matter too much which arm is choose as the optimal.

## 5 Implementation of adaptive design in clinical trials

As indicated earlier, the use of adaptive design methods in clinical trials is very attractive due to its flexibility and efficiency for identifying best clinical benefits of the treatment under investigation. The flexibility, however, may introduce possible operational biases and consequently have an impact on the overall type I error rate. Thus, some practical issues that are commonly encountered when implementing adaptive design methods in clinical trials are necessarily considered to ensure the validity and integrity of the clinical trials. These practical issues are key to the success of the clinical trials utilizing adaptive design methods.

### 5.1 Study planning

Before the implementation of an adaptive design in clinical trials, the following practical issues are necessarily considered. First, the following questions should be addressed in order for determining whether an adaptive design is feasible for the intended trial:

• Will the adaptive design require extraordinary efforts for implementation?

• Are the level of difficulty and the associated cost justifiable for the gain from the adaptive design?

• Will the implementation of the adaptive design delay patient recruitment and prolong the duration of the study?

• Would delayed responses diminish the advantage of the adaptive design?

• How often will the unblinded analyses be practical, and to whom should the data be unblinded?

• How should the impact of a Data Monitoring Committee's (DMC's) decisions regarding the trial (e.g., recommending early stopping or other adaptations due to safety concerns) be considered at the design stage?

Second, the following questions should be addressed to ensure the validity of the adaptive design for the intended trial:

• Will the unblinding cause potential bias in treatment assessment?

• Will the implementation of the adaptive design destroy the randomness?

Third, the following questions regarding the degree of flexibility (e.g., sensitivity or robustness) are necessarily considered:

• Will protocol deviations or violations invalidate the adaptive method?

• How might an unexpected DMC action affect the power and validity of the design?

When designing a trial, we should also consider how to prevent premature release of the interim results to the general public using information masks because releasing information it could affect the rest of the trial and endanger the integrity of the trial. Regarding study design, and we strongly suggest early communication with the regulatory agency and DMC regarding the study protocol and the DMC charter. For broader discussions of planning different adaptive designs, please see the PhRMA full white papers on adaptive design (see, e.g., [16,19,43,46], and [50-52])

### 5.2 Communication with regulatory agency

As indicated earlier, the primary concern when implementing adaptive design methods in clinical trials is its validity and integrity from regulatory perspective. It is a concern whether the application of adaptive design methods may have introduced some unexpected operational bias, which may have an impact on accuracy and reliability of the obtained statistical inference. It is also a concern whether the application of adaptive design methods is able to control the overall type I error rate at the pre-specified level of significance. As a result, regulatory agencies such as the US FDA request strategies or plans for preventing possible operation biases and for controlling the overall type I error rate be developed. In addition, the sponsors are encourage to begin a dialogue about adaptive designs with medical reviewers and/or statistical reviewers from the regulatory agencies as early as possible (preferably at least a year) before the planning of a trial. In a adaptive design conference, an statistician from US FDA office for adaptive design addresses some of the expectations for regulatory submissions utilizing adaptive design([53]). These expectations regarding a clinical trial utilizing adaptive design methods are summarized below:

• It should be prospectively planned;

• It should have valid statistical approaches on modification of design elements that have alpha control and can be defined in terms of ICH E-9 standard;

• It should have valid point estimates and confidence interval estimates;

• It should build on experience from external trials;

• It should takes a *learn and confirm *approach;

• It should have standard operating procedures (SOPs) and infrastructure for adaptive process monitoring to avoid bias;

• It should have SOPs on adaptive design decisions; and

• It should includes documentation of actual monitoring process, extent of compliance, and potential effect on study results.

In order to meet the above expectations, it is critical to communicate with the agency. The advantage for communicating with the regulatory agency early is that the regulatory agency could be of assistance in sharing the data or at least the disease information or models available within the agency. Building off external data and experience is sometimes a crucial element of adaptive design. As indicated in *The Pink Sheet *(December 18 issue, 2006, p. 24), drug development is a knowledge-creating business and the access to external data and experience is extremely helpful when implementing adaptive design methods in clinical trials.

### 5.3 Trial monitoring

In practice, it is recognized that there are often deviations from the study protocol when conducting a clinical trial. It is ethical to monitor the trials to ensure that individual subjects are not exposed, or have limited exposure, to unsafe or ineffective treatment regimens. For this purpose, an independent DMC is usually established. The DMC plays a critical role in monitoring clinical trials. There are common issues that affect a DMC's decision, such as short-term versus long-term treatment effects, early termination philosophies, response to early beneficial trends, response to early unfavorable trends, and response where there are no apparent trends [52,53]. It is recommended that a DMC be established to monitor the trial when an adaptive design is employed in clinical trials, especially when many adaptations are considered for allowing greater flexibility.

#### Stopping rules

The stopping rule chosen in the design phase serves as a (statistical) guideline to the DMC, which will make a decision regarding whether to continue or stop the trial early due to safety, efficacy, or futility [51]. If all aspects of the conduct of the clinical trial adhered exactly to the conditions stipulated during the design phase, the stopping rule obtained during the design phase could be used directly. However, there are usually complicated factors that must be dealt with during the conduct of the trial.

#### Deviation of analysis schedule

DMC meetings are typically based on the availability of its members, which may be different from the schedules set at the design phase. The enrollment may be different from the assumption made at the design phase. Deviation in the analysis schedule will affect the stopping boundaries; therefore, the boundaries should be recalculated based on the actual schedules.

#### Deviation of efficacy variable estimation

The true variability of the response variable is never known, but the actual data collected at interim analysis may show that the initial estimates in the design phase are inaccurate. Deviation in the variability could affect the stopping boundaries. In this case, we may want to know the likelihood of success of the trial based on current data, which is known as conditional and predictive power, and repeated confidence intervals. Similarly, the estimation of treatment difference in response could be different from the initial estimation in the design phase. This could lead to an adaptive design or sample-size re-estimation [12].

#### Safety factors

Efficacy is not the only factor that will affect a DMC's decision. Safety factors are critical for the DMC to make an appropriate recommendation to stop or continue the trial. The term "benefit-risk ratio" is a most commonly used composite criterion to assist in decision-making. In this respect, it is desirable to know the likelihood of success of the trial based on current data, i.e., the conditional power or predictive power. The DMC may also weight the short-term and long-term treatment effects in its recommendations.

Note that the commonly used tools for monitoring a group sequential design are stopping boundaries, conditional and predictive powers, futility index, and repeated confidence interval. These tools can be used in other adaptive designs. Bayesian monitoring tools are appreciated for different adaptive designs.

## 6 Concluding remarks

As indicated earlier, although the use of adaptive design methods in clinical trials is motivated by its flexibility and efficiency, there are still some challenge in implementation. Recently, there are many discussions are around the flexibility, efficiency, validity, integrity, and implementations of various types of adaptive designs. When implementing an adaptive design in a clinical trial, In practice, it is recommended that a couple of principles that (i) adaptation should not alter the quality of trial conduct and (ii) type I error should be preserved must be followed when implementing the adaptive design methods in phase III clinical trials. Following these principles, some basic considerations such as dose/dose regimen, study endpoints, treatment duration, and logistics should be carefully evaluated for feasibility [46]. To maintain the validity and integrity of an adaptive design with complicated adaptations, it is strongly suggested that an independent data monitoring committee (IDMC) should be established. In practice, IDMC has been widely used in group sequential design with adaptations of stopping a trial early and sample size re-estimation. The role and responsibility of an IDMC for a clinical trial using adaptive design should clearly defined. IDMC usually convey very limited information to investigators or sponsors about treatment effects, procedural conventions, and statistical methods with recommendations in order to maintain the validity and integrity of the study.

When applying adaptive design methods in clinical trials, it is suggested that the feasibility of certain adaptations such as changes in study endpoints/hypotheses be carefully evaluated to prevent from any possible misuse and abuse of the adaptive design methods. It should also be noted that although clinical trial simulation does provide "*a*" solution not "*the*" solution for a complicated multiple adaptive design. In practice, "how to validate the assumed predictive model for clinical trial simulation?" is a major challenge to both investigators and biostatisticians.

For a fair and unbiased assessment of treatment effect, the impact of protocol amendments, the major challenges of by design adaptations, and the obstacles of retrospective adaptations prior to database lock and/or unblinding must be carefully evaluated. However, thus far, statistical methodology for addressing these issues (difficulties) are not fully understood and well established, especially under the framework of moving target patient population as the result of protocol amendments. In a recent FDA/PhRMA Workshop on adaptive design held November 2006, the FDA has announced to slow the escalating momentum behind adaptive clinical trial designs to allow time to craft better working definitions of the new models and build a better base before moving forward. As a result, it is suggested that effort should be directed to current issues such as the impact of protocol amendments, challenges in by design adaptations, and obstacles of retrospective adaptations as described in the previous sections.

In summary, from the clinical point of view, adaptive design methods reflect real clinical practice in clinical development. Adaptive design methods are very attractive due to their flexibility and are very useful especially in early clinical development. From the statistical point of view, the use of adaptive methods in clinical trials makes current good statistics practice even more complicated. The validity of the use of adaptive design methods is not well established and fully understood. Guidelines regarding the use of adaptive design methods must be developed so that appropriate statistical methods and statistical software packages can be developed accordingly. Regulatory guidelines can not only prevent possible misuse and/or abuse of adaptive design methods in clinical trials, but also maintain the validity and integrity of the trial.

## Supplementary Material

Additional file 1 Response-adaptive randomization Click here for file
